# Health Safety of Soft Drinks: Contents, Containers, and Microorganisms

**DOI:** 10.1155/2015/128697

**Published:** 2015-01-28

**Authors:** Dorota Kregiel

**Affiliations:** Institute of Fermentation Technology and Microbiology, Faculty of Biotechnology and Food Sciences, Lodz University of Technology, Wolczanska 171/173, 90-924 Lodz, Poland

## Abstract

Soft drinks consumption is still a controversial issue for public health and public policy. Over the years, numerous studies have been conducted into the possible links between soft drink intake and medical problems, the results of which, however, remain highly contested. Nevertheless, as a result, increasing emphasis is being placed on the health properties of soft drinks, by both the industry and the consumers, for example, in the expanding area of functional drinks. Extensive legislation has been put in place to ensure that soft drinks manufacturers conform to established national and international standards. Consumers trust that the soft drinks they buy are safe and their quality is guaranteed. They also expect to be provided with information that can help them to make informed decisions about the purchase of products and that the information on product labels is not false or misleading. This paper provides a broad overview of available scientific knowledge and cites numerous studies on various aspects of soft drinks and their implications for health safety. Particular attention is given to ingredients, including artificial flavorings, colorings, and preservatives and to the lesser known risks of microbiological and chemical contamination during processing and storage.

## 1. Background

**Figure 1:**
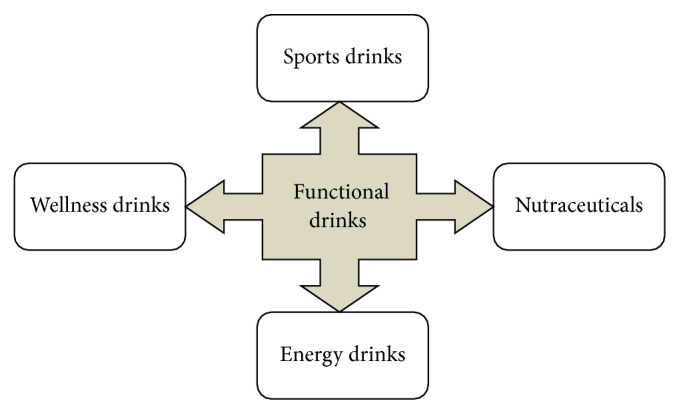
Functional drinks.

Modern soft drinks constitute a diverse group of products. They can be classified in several ways, for example, on the basis of their sugar and fruit juice content, flavoring, carbonation level, main nonwater ingredients, and functionality. Apart from drinking water, the most popular types of soft drinks are as follows: (i) ready-to-drink essence-flavored beverages; (ii) ready-to-drink beverages containing fruits or fruit juice; (iii) beverages ready-to-drink after dilution [1] (Table 1). Functional drinks are a rapidly growing subsector of the market and include drinks enriched with juices, vitamins, and minerals; sports and energy drinks; wellness drinks and nutraceuticals. Many functional drinks have been developed to provide specific medical or health benefits, such as promoting heart health, improving immunity and digestion, and helping to boost energy [2] (Figure 1). The target markets for functional beverages are diverse, and products are often tailored towards particular target markets, for instance, according to age and gender, with a growing focus on children, women, and seniors.

## 2. Legislation

Soft drinks intended for human consumption are covered by national regulations based on codes and standards [3]. For example, the Canadian Food and Drug Regulations prescribe quality, composition, and labelling standards which also apply to nonalcoholic beverage manufacturers [4]. Japanese legislation includes the Food Sanitation Act for food additives and labeling of processed foods. Japan also has a positive list of additives [5]. In Australia and New Zealand, Standard 2.6.2, Nonalcoholic Beverages and Brewed Soft Drinks, defines a number of products and sets certain compositional requirements for packaged water, electrolyte drinks, brewed soft drinks, and beverages [6]. In the United States, soft drinks are regulated by the Food and Drug Administration (FDA). Sections 401 (Food Standards) and 409 (Food Additives) regulate additives listed on the Food Additives Status List, formerly called Appendix  A of the Investigations Operations Manual (IOM), which includes those additives specified under the Federal Food, Drug, and Cosmetic Act [7]. Soft drinks ingredients must comply with all applicable FDA safety requirements [8].

In the EU, beverages are subject to EU legislation on microbiological criteria, food additives, and general hygiene requirements for the production, storage, and trade of food products [9, 10]. The four main EU regulations are as follows: (i) Regulation EC 1331/2008, which describes a common authorization procedure for food additives, enzymes, and flavorings; (ii) Regulation EC 1332/2008 on food enzymes; (iii) Regulation EC 1333/2008 on food additives, which lists (in Annexes II and III) approved food additives together with the food categories with which they may be used; and (iv) Regulation EC 1334/2008 on flavorings. Together, these regulations are often referred to as the “Package on Food Improvement Agents” (FIAP) [11]. In 2011, the FIAP was supplemented with the following new regulations: (i) Regulation EC 1129/2011, amending Annex II to Regulation (EC) number 1333/2008 on food additives; (ii) Regulation 1130/2011, amending Annex III to Regulation (EC) number 1333/2008 on food additives, food enzymes, food flavorings, and nutrients; and (iii) Regulation EC 1131/2011 amending Annex II to Regulation (EC) number 1333/2008 with regard to steviol glycosides. In addition, a special program to evaluate food additives was established, set up by Regulation EC 257/2010. According to this regulation, the reevaluation of approved food additives must be completed by the end of (i) 2015 for food colors; (ii) 2018 for all additives other than colors and sweeteners; (iii) 2020 for food sweeteners.

## 3. Ingredients

Soft drinks typically contain water, sweetener (8 ÷ 12%, w/v), carbon dioxide (0.3 ÷ 0.6% w/v), acidulants (0.05 ÷ 0.3% w/v), flavorings (0.1 ÷ 0.5% w/v), colorings (0 ÷ 70 ppm), chemical preservatives (lawful limits), antioxidants (<100 ppm), and/or foaming agents (e.g., saponins up to 200 mg/mL). Some types of soft drink use sugar substitutes. However, certain ingredients may be hazardous to health if consumed in large quantities, and there is widespread concern generally with regard to preservatives and sweeteners. Therefore, while on the one hand there is a trend to produce ever wider ranges of more specialist soft drinks, there is also pressure to minimize the use of, in particular, artificial and synthetic additives and ingredients.

### 3.1. Water

Conventional soft drinks contain 90 percent water, while diet soft drinks may contain up to 99% water. Drinking water includes trace amounts of various ions which alter its taste. Soft drinks manufacturers usually use softened water to prevent off-tastes from chlorine residues [12]. The most frequent methods of removing water hardness employ ion-exchange polymers or reverse osmosis. Other approaches include precipitation methods and sequestration using chelating agents. These procedures reduce the concentration of metal ions to approximately 50 ppm Mg and Ca [13]. Water for soft drinks should fulfill the physical, chemical, and microbiological criteria for drinking water according to European Directive EC 98/1983, US Environmental Protection Agency (EPA), and World Health Organisation (WHO) standards [14].

### 3.2. Sugars and Sweeteners

With the exception of zero calorie products, soft drinks usually contain between 1% and 12% sugar (w/w). Sucrose, glucose, or fructose, in various forms, is used as natural carbohydrate sweeteners. The most common natural sweeteners provide glucose, the primary source of energy. Sucrose (saccharose) is a disaccharide composed of glucose and fructose molecules bound by an *α*-1,2 linkage. This sugar can preserve and enhance the flavor of a drink and gives a satisfying sensation. Other natural carbohydrate sweeteners are as follows: trehalose, isomaltulose (Palatinose), and D-tagatose [12]. Trehalose is a disaccharide composed of two glucose molecules bound by an *α*-1,1-linkage. This compound is characterized by high thermostability and a wide pH stability range. Its relative sweetness is around 45% that of sucrose. The metabolism of trehalose is similar to other disaccharides: Ingested trehalose is hydrolysed into glucose and absorbed in the small intestine [15]. Isomaltulose, like saccharose, is a disaccharide of glucose and fructose, but in contrast to sucrose is joined by an *α*-1,6 glycosidic bond. Isomaltulose is a tooth-friendly disaccharide with slow energy release, low glycemic index, and mild sweetness [16]. Tagatose has a structure similar to that of fructose. It is almost as sweet as sucrose and has flavor-enhancing properties. Most ingested tagatose is fermented by colon microflora, resulting in the production of short-chain fatty acids, which are then absorbed almost completely and metabolized.

As is well known, overconsumption of sugars can cause negative health effects, such as obesity, diabetes mellitus, or nonalcoholic fatty liver disease. Natural sweeteners provide 1.5 ÷ 4.0 calories per gram and have been linked with weight gain [17]. Fructose contributes to the formation of advanced glycation end products, which may be factors in the onset of diabetes, hasten aging processes, and cause thickening of artery walls [18]. Since tagatose is absorbed slowly and only incompletely in the intestine, consuming excessive amounts may lead to flatulence and/or laxation [13].

Due to health, including dental health, concerns, alternative sweeteners are commonly added to soft drinks which are produced and labeled as containing “no added sugar” [18]. Most low-calorie beverages contain intense sweeteners, which have been approved for use within levels of Acceptable Daily Intake (ADI) and in accordance with the appropriate regulations. The most commonly used sweeteners (with maximum permitted dosage in the EU) are aspartame (600 mg/L), acesulfame K (350 mg/L), sucralose (300 mg/L), and saccharin (80 mg/L) [17].

Aspartame (E951) consists of two amino acids: L-phenylalanine and L-aspartic acid, esterified to methyl alcohol. This compound is 200 times sweeter than sucrose and leaves no unpleasant aftertaste. However, it is unstable at high temperatures and therefore is unsuitable for use in pasteurized beverages. Aspartame is also unstable in aqueous solutions, where it is gradually converted into diketopiperazine (DKP). In the body, aspartame is broken down into phenylalanine (about 50% by weight), aspartic acid (40%), and methanol (10%). Soft drinks with aspartame must carry a label indicating that the product contains phenylalanine, which can be harmful to individuals with phenylketonuria, who must strictly limit the intake of this amino acid. Aspartame is permitted in more than 100 countries in the world. Authorities that have approved aspartame include the FDA, the Agence Française de Sécurité Sanitaire des Aliments (AFSSA), and the Joint FAO/WHO Expert Committee on Food Additives (JECFA). In 2013, the European Food Safety Authority (EFSA) approved aspartame for use in food and beverages [19].

Acesulfame K (E950) is 200 times sweeter than sucrose, thermo- and pH-stable, and freely soluble in water. This compound is neither metabolized nor stored in the body. The FDA, FAO/WHO, JECFA, and the Scientific Committee on Food of the European Union (SCF) have concluded that acesulfame is safe for use in foods and beverages.

Sucralose (E955) is derived from sucrose through selective replacement by chlorine atoms of three hydroxyl groups. This compound is 600 times sweeter than sucrose but has no calories. Readily soluble in water and acid solutions, sucralose hydrolyses slowly to its monosaccharides. Sucralose has been determined safe by FAO/WHO, JECFA, and FDA and is permitted for use in beverages in more than 40 countries, including the United States, Canada, Australia, and Mexico [20]. In 2011, sucralose, along with other sugar substitutes, was cleared for use by the EFSA as a sweetener in food and beverages [21].

Saccharin (E954) is 300 times sweeter than sucrose but leaves a bitter/metallic aftertaste. Use of saccharin in foods dates back to 1907. This sweetener is permitted in more than 100 countries around the world [13, 15].

Other less common sweeteners include thaumatin (E957) and stevioside (E960). Thaumatin is a mixture of proteins isolated from the katemfe fruit (*Thaumatococcus daniellii* Benth) from West Africa. It is the most powerful natural sweetener, 2000 times sweeter than sugar. It is used in food as a safe sweetener and flavour modifier [12]. Stevioside is another intense sweetener, 200 times sweeter than sucrose, extracted from the leaves of the stevia plant (*Stevia rebaudiana* Bertoni). It has a long history of use in several countries, including Japan and Paraguay [15]. Stevioside is permitted for use in many countries, including the USA, France, Mexico, Korea, Taiwan, China, Russia, Australia, Argentina, New Zealand, Colombia, Peru, Uruguay, Brazil, Switzerland, and Malaysia. In Canada, stevia extract is sold as a natural health product. In Europe (except France), stevia is permitted as a dietary supplement but is not yet permitted for use as a sweetener in food and beverages [22]. In France, stevia extract (rebaudioside A) is permitted for use as a sweetener in foods and beverages [23]. Other sweeteners used more rarely in soft drinks include cyclamate, erythritol, and neotame [17]. Over the years, the number of available sweeteners has steadily increased.

### 3.3. Fruit Juices

Fruits and fruit juices are a rich source of various nutrients and bioactive compounds, such as fiber, sugars, organic acids, phosphates, minerals, and vitamins, as well as colors, flavors, and antioxidants [13]. The sugar content in natural fruit juices varies depending on the type of fruit. All fruit juices contain fructose but vary in their amounts of sucrose, glucose, and sorbitol. Lemons, limes, rhubarb, raspberries, blackberries, and cranberries are relatively low in sugars. For example, one tablespoon of fresh lemon juice has 4 calories. Fruit groups high in saccharides include grapes, tangerines, cherries, pomegranates, mangoes, figs, and bananas. About 12 medium-size grapes contain 52 calories [18].

Apart from sugars, vitamins, and minerals, fruits and fruit juices contain another important component of a balanced diet: fiber. Dietary fiber is defined as nondigestible carbohydrates and lignin, including starch or the nonstarch polysaccharides cellulose, hemicelluloses, pectins, hydrocolloids, and oligosaccharides. It has been confirmed that high-fiber diets can improve digestive health and help prevent heart disease, diabetes, weight gain, and some cancers [24]. Fruits rich in fiber include apples, blackberries, pears, and raspberries. One medium apple with its skin contains 4.4 grams of fiber, half a cup of fresh blackberries contain 3.8 grams, and a half-cup of raspberries contains 4 grams, while the same size serving of strawberries provides only 2 grams of dietary fiber. According to the US Department of Agriculture, regardless of age, weight, or gender, people should consume at least 14 grams of fiber for every 1 000 calories [25]. The EFSA recommends average dietary fiber intakes of 10 to 20 g per day for young children (<10 to 12 years), 15 to 30 g per day for adolescents, and 16 to 29 g per day for adults [26].

### 3.4. Acidity Regulators and Carbon Dioxide

The carbonation of soft drinks varies from 1.5 to 5 g/L. Carbon dioxide is supplied to soft drinks manufacturers either in solid form (as dry ice) or in liquid form maintained under high pressure in heavy steel containers. This process makes the drink more acidic, which serves to sharpen the flavor and taste [27]. It also helps preserve soft drinks for longer time [28]. Acidity regulators are used in soft drinks to improve their taste by balancing the sweetness. Human saliva is almost neutral (pH approximately 6.8), and when our taste receptors interact with acids in food or drink this sensation is perceived as sourness [29]. Acids also play an important role in the natural preservation of soft drinks [30].

In most of beverages, citric acid (E 330) is the first choice for use as an acid regulator, as it has several additional benefits, such as enhancing the activity of beneficial antioxidants and adding aroma. Malic acid (E 296) is used when strong flavor enhancement is required and mostly in combination with citric acid. Succinic acid (E 363) has a wide range of applications, including dairy and desserts, but due to regulations it can only be used in instant beverages to be prepared at home [27]. Citric acid, succinic acid, and phosphoric acid are all subject to EC Regulation 1333/2008 on Food Additives.

Phosphoric acid (E 338) has a strong effect on pH and is commonly used to give a specific taste profile to cola-type beverages. Use of phosphoric acid remains controversial, since it has been associated with adverse health effects. High levels of phosphorus in the blood, referred to as “hyperphosphatemia,” can lead to organ damage, most notably of the kidneys. Poor kidney function can raise levels of phosphorus in the blood, which in turn lowers calcium levels, increasing the risk of brittle bone disease. Moreover, increased serum phosphorus levels, as well as other mineral abnormalities, can individually and collectively contribute to vascular calcification and cardiovascular disease [31–33]. The EFSA has presented its opinion on the upper intake level of phosphorus, according to which healthy individuals can tolerate up to 3000 mg/day without negative effects. However, in some individuals mild gastrointestinal symptoms have been reported with supplemental intakes of >750 mg phosphorus per day [34]. Phosphoric acid and its derivatives were permitted in the European Union prior to 2009 and are therefore included in the program for reevaluation of food additives [35].

### 3.5. Flavorings and Colorings

The use of colorings in soft drinks serves several important functions: (i) making the product more aesthetically appealing; (ii) helping to correct for natural variations in color or for changes during processing or storage; (iii) contributing to maintaining the qualities by which the drink is recognized. There are three basic categories of colorings: natural colors, artificial colors, and caramels. Natural colorings can be extracted from plants, fruits, and vegetables and comprise two main categories: (i) yellow to orange carotenoids, extracted from plants; (ii) bright red to purple anthocyanins obtained commercially from a range of fruits and vegetables. Natural colorings are also added to soft drinks for their antioxidant properties [36]. Due to increasing consumer preference for natural colors, the trend in both the EU and the US markets in recent years has been for manufacturers and retailers to reduce the use of artificial colors in their products.

The use of colorings in EU countries must comply with Food Additives Regulation (EC) 1333/2008. All permitted colors are listed in Annex II and Annex III with limitations specified on their use. The EFSA's expert Scientific Panel which deals with food additives, the ANS Panel, has begun a reassessment of all permitted food colorings. For example, in 2013 the EFSA recommended that new tests be carried out to address the possible genotoxicity of Allura Red AC and other “sulphonated monoazo dyes”: Amaranth, Ponceau 4R, Sunset Yellow FCF, Tartrazine, and Azorubine/Carmoisine [37]. Another coloring, Ponceau 4R (E124), is an allergen, which may elicit intolerance in people allergic to salicylates (aspirin). It is also a histamine liberator, which may intensify symptoms of asthma. Ponceau 4R is even considered carcinogenic in some countries, including the USA, Norway, and Finland. It is currently on the Food and Drug Administration's (FDA) list of banned substances in the USA. The EFSA has decided to decrease the ADI for Ponceau 4R from 4 mg/kg to 0.7 mg/kg bodyweight per day [38].

Flavorings, in contrast to colorings, are used in comparatively small amounts, so consumer exposure is relatively low. European Union legislation defines the different types of flavorings as natural, natural-identical, and artificial. Based on work by the EFSA, in 2012 the European Commission established a list of flavorings which may be used in the EU. The EFSA has also developed guidelines which specify the data the industry should submit in order to facilitate the safety evaluation of new flavorings [39].

### 3.6. Preservatives

Chemical preservatives are used to improve the microbiological stability of soft drinks. The types of chemical preservatives that can be used depend on the chemical and physical properties of both the preservative and the beverage. The pH of the product, the presence of vitamins, the packaging, and the conditions of storage will determine what types of preservative, if any, should be used to prevent microbial growth. Sorbates (E 200–203), benzoates (E 210–213), and dimethyl dicarbonate (DMDC) (E 242) are permitted in ready-to-drink beverages in Europe.

Sorbates are very effective preservatives against bacteria, yeasts, and molds. The antimicrobial effectiveness of sorbates depends on the physical and chemical properties of the beverage. Sorbates and benzoates are often used in combination, especially in highly acidic drinks [40]. Sorbic acid affects yeast growth by inhibiting the uptake of amino acids and the function of sulfhydryl enzymes, while benzoic acid destroys the internal proton level of microbial cells [41].

Benzoic acid occurs naturally, notably in cranberries, cinnamon, plums, and currants and has been used to inhibit microbial growth for many years, including nonalcoholic beverages. Benzoate salts are particularly well suited for use in carbonated, nonalcoholic, and juice beverages. They are more stable than benzoic acid, more soluble in water, and work best at pH levels between 2 and 4.4. According to Battey et al. [41], the three significant growth predictors for spoilage yeasts are the variables: pH level, potassium sorbate, and sodium benzoate. However, benzoates react with ascorbic acid (vitamin C) and form benzene, especially if they are stored for extended periods at high temperatures. In the United States, the EPA has classified benzene as a known human carcinogen for all routes of exposure [42]. Despite the fact that producers have developed methods to prevent or minimize its occurrence, and the frequency and levels of benzene formation in soft drinks have not represented a risk to public health in the past, benzoates are being used more rarely in the industry. This is partly due to new processing techniques, which have reduced the need to use benzoates in soft drinks production. However, these preservatives are still necessary to maintain quality in some beverages.

DMDC is commonly used as a preservative in cold-sterilized soft drinks. DMDC is very reactive and rapidly breaks down when added to a substrate, such as a water-based beverage. The principal products are methanol and carbon dioxide; however, the methanol concentrations after treatment with DMDC are of no toxicological concern. DMDC performs a broad range of antimicrobial actions against yeasts, mould fungi, and bacteria. Dimethyl dicarbonate penetrates into the cell and deactivates enzymes, leading to the destruction of the microorganism [43]. Various international regulatory bodies have evaluated DMDC and concluded that there are no health or safety concerns when this preservative is used in permitted food categories at prescribed usage levels. The safety of DMDC was evaluated by the USFDA in 1988 and approved for use in wines as a yeast inhibitor up to a concentration of 200 mg/L. The European Scientific Committee on Food evaluated DMDC in 1990 and concluded that it was suitable for the cold sterilisation of soft drinks and fruit juices at levels up to 250 mg/L. DMDC was also evaluated in 1990 by the Joint FAO/WHO Expert Committee on Food Additives. It was considered acceptable as a cold sterilising agent for beverages when used in accordance with Good Manufacturing Practice up to a maximum concentration of 250 mg/L [44].

Sulfites are sulfur-containing compounds and have been used for centuries to reduce or prevent spoilage and to enhance the flavor or preserve freshness in fruit juices. However, in recent years sulfites, as well as sorbates and benzoates, have been associated with allergic reactions in some people, especially sufferers of asthma [12]. The FDA requires labeling on any food containing a sulfite concentration of more than 10 parts per million (ppm). EU legislation also states that the additives used in a product should be listed on the label so as to permit consumers to make informed choices and avoid certain additives when necessary [40].

The use of natural antimicrobial compounds in soft drinks has become a subject of concern for both consumers and industry. This is due to two primary factors. Firstly, the misuse and mishandling of various preservatives have resulted in a remarkable rise in the number of microorganisms, including some foodborne pathogens, which are more tolerant to food processing and preservation methods. Secondly, increasing consumer awareness of the potential negative impact of synthetic preservatives on health, in contrast to the benefits of natural additives, has generated interest in the development and use of natural products. Compounds derived from natural sources have antimicrobial properties against a broad range of foodborne pathogens and could be used as alternative preservatives, with the potential of enhancing the safety and quality of drinks [45].

Since the first scientific experiments on plant antimicrobial activity and chemical composition were documented in the second half of the 19th century, a wide range of food plants, including various berries and fruits, have been shown to inhibit microbial growth. Different plants belonging to various families possess strong antimicrobial properties. For example, the Rosaceae family includes several species which are well known for their antibacterial activity, such as cloudberries (*Rubus chamaemorus*), raspberries (*Rubus idaeus*), strawberries (*Fragaria ananassa*), blackberries (*Rubus fruticosus*), chokeberries (*Aronia melanocarpa*), and European rowan (*Sorbus aucuparia*) [46, 47]. The list of plants which exhibit antimicrobial effects is long, over 1000 fruits and herbs [48].

The antimicrobial activity of plant extracts is based on phenolics (simple phenols, phenolic acids, quinones, flavones, flavonoids, flavonols, tannins, and coumarins), terpenoids and essential oils, alkaloids, lectins, polypeptides, and so forth [49]. Various aroma compounds and citron essential oils containing citral, *β*-pinene, limonene, linalool, and *α*-pinene, combined with mild heat treatment, have been used to inhibit the growth of* S. cerevisiae* in noncarbonated soft drinks. Clary, sage, juniper, lemon, and marjoram essential oils were shown by Lucera et al. to preserve apple juice [50]. The antimicrobial effects of these essential oils have been observed in the acidic pH range. Synergism or additive effects may result from the combination of different active compounds. Although some active agents are known to influence smell or taste, this has rarely been the object of close study. One solution to the problem may be to use combinations of different preservation systems, which would provide the benefits of each while at the same time appreciably reducing the amount of antimicrobial agent required.

### 3.7. Other Ingredients

Various hydrocolloids, such as guar and locust gum, pectin, and xanthan, are used as stabilizers and thickeners, especially in diet drinks (to improve mouthfeel) and fruit juice drinks (to reduce phase separation). Antioxidants, most commonly ascorbic acid, are used to prevent the deterioration of flavors and colors, especially when drinks are packaged in oxygen-permeable bottles and cartons. Functional drinks, nutraceuticals, and wellness drinks contain a variety of unconventional ingredients, which can include the so-called “superfruits” (e.g., pomegranate, acai, acerola, noni, and mangosteen), berries (e.g., cranberries, blackberries, strawberries, and blueberries), or botanical extracts (e.g., ginger, ginkgo, and melissa) [36]. Some functional drinks use plant sterols and omega-3 fatty acids to promote heart health. Others include dietary fibres, such as inulin and maltodextrin, which are prebiotics that selectively modulate host microbiota, conferring health benefits [51].

The most common active ingredients of energy drinks are taurine (average 3180 mg/L), caffeine (360–630 mg/L), and caffeine-rich plant extracts (e.g., tea, ginseng, guarana, and yerba mate). Energy drinks also contain B-vitamins (B3, B6, and B12) [52]. The main ingredients in sport drinks are carbohydrates in the form of glucose, fructose, and maltodextrin (5.5–8.2% w/v), salts, and water [53]. Typical sodium and potassium concentrations are 20 ÷ 30 and 5 mM, respectively [54]. In addition, an increasing number of other functional ingredients are being used in sports drinks. Energy drinks are promoted for their stimulating effects and claim to offer a variety of benefits including increased attention, endurance, and performance. They are also associated with weight loss and entertainment. However, there are increasing reports of caffeine intoxication from energy drinks. In children and adolescents, who are not habitual caffeine users, vulnerability to caffeine intoxication may be markedly increased due to an absence of pharmacological tolerance. Several studies suggest that energy drinks may serve as a gateway to other forms of drug dependence [55].

Cereal-based high-fibre ingredients are another area of interest. The health benefits of *β*-glucans and cereal fibres in general are well known. The relationship between consuming *β*-glucans and healthy levels of blood cholesterol has been confirmed by the EFSA [56]. Based on the evidence, the EFSA Panel concluded that foods, with at least 3 g/d of *β*-glucans from oats, oat bran, barley, and barley bran, or with mixtures of nonprocessed or minimally processed *β*-glucans, can reduce blood cholesterol in adults with normal or mildly elevated cholesterol levels.

### 3.8. Probiotic Bacteria

The market for probiotic foods and beverages is expanding rapidly. In the last decade, five hundred new probiotic food and beverages have been introduced around the world. Europe is currently the largest probiotics market, due to widespread public awareness of the benefits of probiotic yogurts and fermented milk. Drinking yogurt is a popular soft drink in Europe and the USA. Yakult (Japan, China), tibicos (tibi) (Caucasus), kombucha (China, Korea, Japan, Russia), and kvass (Russia) are further examples of fermented beverages of various origins. Probiotic lines of frozen yogurt, fruit/vegetable juices, or soya-based drinks are now beginning to appear on the market [57]. The World Health Organization states that “probiotics…should not only be capable of surviving passage through the digestive tract, by exhibiting bile and acid tolerance, but also should have the capability to proliferate in the gut.” The most common probiotics, used in the production of probiotic milk products, belong to* Lactobacillus plantarum*,* Lactobacillus rhamnosus, Lactobacillus paracasei, Bifidobacterium lactis, Lactobacillus acidophilus*, and* Pediococcus pentosaceus*.

Unfortunately, many commercial products have been pasteurized or preserved chemically, destroying the bacteria. Probiotic bacteria also do not survive well in natural fruit or vegetable juice environments. The viable cell counts of lactic cultures in fermented juice gradually decrease during the first few weeks of cold storage [58, 59]. In research conducted by Mousavi et al. [60], it has been shown that in probiotic pomegranate juice* L. plantarum* and* L. delbrueckii* have higher viability than* L. paracasei* and* L. acidophilus* during storage. Considerable amounts of citric acid, a major organic acid in pomegranate juice, were consumed by all probiotic lactic acid bacteria. Viable bacterial cells remained at their maximum level for 2 weeks but decreased dramatically after 4 weeks. However, it was found that the additives vitamin C, grape extract, and green tea extract improved rates of survival by probiotic bacteria. Three samples of a model juice containing grape seed extract, green tea extract, or vitamin C were given the same initial population of 8 log CFU/mL probiotic bacteria and at the end of a 6-week storage period had average viabilities of 4 log CFU/mL, 7 log CFU/mL, and 6 log CFU/mL, respectively [58].

## 4. Bottle Material

Harmonization of legislation on food contact material (FCM), meaning that for specific food contact materials the EU fixes specific requirements which allow them to be considered to be generally in compliance with the rules in operation in all EU territories, began in the early 2000s and has since been in a process of constant evolution. Currently, EU legislation covers the following FCM: cellulose films, ceramics, plastics, elastomers and rubbers, coatings, and adhesives. The general requirements for all food contact materials are laid down in Framework Regulation EC 1935/2004. Any migration of unsafe levels of chemical substances from the material to the food, changing the composition of the food in an unacceptable way or having adverse effects on its taste and/or off-flavor, must be prevented. Good Manufacturing Practice for materials and articles intended for contact with food is described in Regulation EC 2023/2006. There are also EU regulations and directives for specific materials: ceramics, regenerated cellulose film, plastics, recycled plastics, and active and intelligent materials. Some directives cover single substances or groups of substances used in the manufacture of food contact materials.

It would be difficult to present a complete introduction to US Food and Drug Administration (FDA) legislation and its practical use in the clearance of food contact materials, a task which is in any case beyond the scope of this paper. Central to its policy, however, is the FDA's “Threshold of Regulation” (TOR) program, which sets limits under which certain substances that may migrate into foods from packaging or food processing equipment do not need to be included as additives on product information. Over 3000 of these substances, known as “indirect” food additives, are listed along with administrative and chemical information as part of the Priority-Based Assessment of Food Additives (PAFA) program. These include components in adhesives, coatings, paper, and paperboard, as well as polymers, adjuvants, and production aids. Additional exemptions for substances are listed in separate inventories. Title 21 of the Code of Federal Regulations (CFR), part 170,39, “Threshold of Regulation for Substances Used in Food-Contact Articles,” presents conditions and guidelines for making a TOR request, which producers must submit in order to ensure that indirect food additives and their estimated exposure qualify for exemption under the TOR process [61–63].

Most convenience beverage bottles in both the United States and the European Union are made from glass and plastics. Seven resin codes (usually found on the bottom of containers and bottles) are used to indicate the types of resin in plastics. Of these, the most commonly used in the food industry are (i) polyethylene terephthalate (PET) for soft drink bottles and water bottles; (ii) low density polyethylene (LDPE) for wrapping films and grocery bags; (iii) polypropylene (PP) for syrup bottles, yogurt tubs, and bottle caps; (iv) polystyrene (PS) for single-use coffee cups. Soft drinks are also packaged in tin-free steel, aluminum, treated cardboard cartons, or foil pouches.

Glass bottles seem to be the safest option for food packaging and storage, since there is no possible transferal of contaminating chemicals. Glass recycling is also more environmentally friendly than plastic recycling, which can release toxic chemicals. However, soft drinks containers are often stored under unpredictable conditions for several months before consumption, and this can have an effect on how they interact with the liquids inside. Different materials (glass, soft PET, and hard PET) and different bottle colors have different effects. It has been demonstrated that under certain conditions glass bottles can contaminate bottled water with Pb and Zr [64]. Other elements may be added to glass during the production process to determine color (Fe, Cr for green colors, Co for blue colors). Reimann et al. [65] have shown that many more elements leach from glass than from PET bottles. Comparing water sold in PET-bottles to the same water sold in glass bottles, Ce, Pb, Al, and Zr were found to leach most from glass, but levels of Ti, Th, La, Pr, Fe, Zn, Nd, Sn, Cr, Tb, Er, Gd, Bi, Sm, Y, Lu, Yb, Tm, Nb, and Cu were all also significantly higher compared to water sold in PET bottles. The pH of beverages can also be assumed to play an important role in the release of elements from their containers. In most cases leaching increases considerably at a lowered pH of 3.5, often by a factor 10 or more. The leaching of chemical elements from mineral water bottle materials was also found by these authors to be temperature-dependent [66].

Polyethylene terephthalate (PET) bottles have been shown to contaminate water with Sb, with concentrations increasing with storage time [67–69]. It is also well known that acetaldehyde and antimony leach from PET bottles, although this process is not fully understood due to the number of contributing factors (contact time, type of simulant, temperature, exposure to sunlight, and bottle color). In light of these difficulties, further investigation is needed to understand the migration of degradation products from PET and ensure that it is safe for use in food and drinks containers [70].

Plastic bottles have other chemicals that can contaminate the beverages they contain. One example is bisphenol A (BPA) which can affect the natural communication system of hormones when ingested. Phthalates also leach into the bottled water we drink after as little as 10 weeks of storage, or much faster if the bottles have been left in the sun. Phthalates are used as plasticizers to increase the flexibility of plastics, including PVC. As they are not chemically bound in plastics, they can leach into the environment. Moreover, phthalates are lipophilic compounds and have been found to bioaccumulate in fats. Exposure to phthalates can be detrimental to human health. The larger molecular weight phthalates, di(2-ethyl-hexyl) phthalate (DEHP), di-n-butyl phthalate (DBP), diisononyl phthalate (DiNP), are suspected carcinogens and are known to be toxic to the liver, kidneys, and reproductive organs [71]. More toxic chemicals leach from reused plastic bottles than from new.

The bottle material may also influence the number and type of microorganisms in soft drinks, due to cells adhering to the bottle surface. Plastic bottles have higher surface roughness, hydrophobicity, and electrostatic charges than glass bottles and usually have higher microbial counts [72]. Nutrients from soft drinks are absorbed by and concentrate on plastic surfaces and therefore are more available to bacteria. Adsorption of organic matter is then the basis for adhesion of microorganisms to bottle surfaces. Jayasekara and coworkers [73] report considerable variation between bottles from the same water producer and found that up to 83% of the total microbial population adhered to the inner surfaces of those bottles. In contrast, Jones et al. [72] detected much lower levels of adhesion. Their studies using scanning electron microscopy revealed sparse cell adherence to the surfaces of polyethylene terephthalate (PET) packaging, while biofilms represented around 0.03–1.79% of the total viable count in 1.5 L water bottles. The differing results of these studies suggest that bottles made from different resins may provide microhabitats suitable for specific microflora [74].

## 5. Microbial Spoilage

Microbial contamination of soft drinks usually originates during the production process. The raw materials, factory environment, microbiological state of the equipment and packages, and lack of hygiene are all possible factors [75, 76]. Packaging materials such as cans and bottles can also be sources of contamination.

There are two main methods of producing soft beverages. In the first, the syrup is diluted with water, after which the product is cooled, carbonated, and bottled. In the second, a precise amount of syrup is measured into each bottle, which is then filled with carbonated water. The processes of blending of syrups and mixing with water, container washing, and container filling are all performed almost entirely by automatic machinery. Returnable bottles are washed in hot alkaline solutions for a minimum of five minutes and then rinsed thoroughly. Single-use containers are usually air- or water-rinsed before filling. The preparation of noncarbonated beverages requires similar processes. However, since they lack the protection against spoilage provided by carbonation, noncarbonated drinks are usually pasteurized, either in bulk, by continuous flash pasteurization prior to filling, or in the bottle. Cold aseptic filling is usually used, especially for sport drinks, teas, flavored waters, and juices.

The condition of raw materials and the production environment can directly or indirectly affect the safety of manufactured soft drinks. The following obligatory systems have been designed to ensure food safety: Good Manufacturing Practice (GMP), Good Hygienic Practice (GHP), and Hazard Analysis and Critical Control Point (HACCP) [77]. However, when technological processes go wrong, the method of beverage production will have a significant impact on the type of spoilage microflora.

Spoilage is a metabolic process that causes beverages to be undesirable or unacceptable for human consumption, due to changes in sensory characteristics. Microbial contamination of raw materials can cause odors, gushing, and other undesirable defects in the final product. Soft drinks are high in water activity and often rich in vitamins and minerals, so they present an attractive environment for microorganism [75, 78–81]. Soft drinks can contain different types of microorganism, but aciduric microorganisms are the only significant spoilage microflora; although new, sometimes exotic ingredients used in soft drinks could introduce additional spoilage species. The most important microorganisms with their typical effects on contaminated soft drinks are presented in Table 2.

### 5.1. Yeasts

Yeasts are considered to be the primary spoilage microbes in carbonated products, mainly due to their ability to resist high carbonation and low pH levels.* Zygosaccharomyces bailii* yeast can tolerate moderately high carbonation and is also able to grow at refrigerator temperatures [82]. Other fermentative yeasts isolated from spoiled soft drinks include* Saccharomyces, Brettanomyces, Hanseniaspora, Hansenula*, and* Pichia*. Most species grow within a pH range from 1.5 to 8.5 with optimal growth in the range of 3.0 ÷ 6.5 [79, 83]. Yeasts produce ethanol as an end-product of fermentation and the ethanol level in spoiled soft drinks may exceed the legal limit for nonalcoholic products. Fermentation can also result in bulging cans or exploding bottles. CO_2_ formation by fermentative spoilage yeasts, measured after 2 weeks of growth in a soft drink containing 1 M glucose, generated 2 to 7 bars of gas pressure [75]. Spoilage yeasts can also alter the beverage by changing its pH or degrading preservatives. For example, the spoilage yeast* Saccharomyces cerevisiae* is able to degrade sorbic acid to form a volatile hydrocarbon, 1,3-pentadiene [84]. The flavor of pentadiene is described as kerosene-like with additional notes of plastic, paint, and geranium, depending on the concentration. Some spoilage yeasts also possess lipolytic enzymes which can degrade the fatty acids [41].

A special group of weak-fermentative yeasts causes spoilage when production processes go wrong. These belong to* Candida davenportii*,* C. parapsilosis,* or* Debaryomyces* spp. Some yeasts can serve as indicators of poor hygiene in manufacturing plants but do not cause spoilage in the final product. These include red aerobic yeasts* Rhodotorula, Sporidiobolus,* and* Sporobolomyces *and the black genus* Aureobasidium*. These yeasts usually predominate in soft drinks factories and are typically found on technological surfaces in places difficult to clean and disinfect [75, 85].

### 5.2. Molds

Molds grow as white, delicate, fluffy, cottony masses suspended in soft drinks. Fungal spores or conidia and mycelium fragments can contaminate beverages at any stage of the production process [86]. Like yeasts, molds can become established in manufacturing factories due to poor process hygiene or spread from contaminated packages. Water availability and high acidity are basic conditions for fungal spoilage of soft drinks. The maximum and minimum value of pH for growth are dependent on other factors such as water activity, temperature, or the type of acid used. For example, citric, phosphoric, and tartaric acids promote growth at lower pH, when acetic or lactic acids are present [87]. Unlike yeasts, however, molds require oxygen, although some species can grow at low oxygen concentrations (~0.01% v/v) [86, 88]. Mold spores cannot grow in carbonated beverages but can survive.* Aspergillus, Penicillium, Rhizopus, Cladosporium,* and* Fusarium* are often detected in soft drinks and in factory environments [89]. They produce pectinases, resulting in undesirable changes in taste and flavor, and less frequently gas formation [89–91]. Fungal contamination may also lead to discoloration, formation of allergens, and the production of toxic compounds [88]. Mycotoxins are fungal metabolites which cause sickness or death in people and animals when ingested, inhaled, or absorbed [92]. When present in high levels, mycotoxins can have toxic effects ranging from the acute (e.g., kidney or liver damage) to the chronic (increased cancer risk and suppressed immune system). Production of a particular mycotoxin is a species- or strain-specific property. The relevant mycotoxins related to foods and beverages are produced by species in the genera* Aspergillus, Penicillium, Fusarium,* and* Alternaria* and include aflatoxins, ochratoxin A, patulin, and* Fusarium* toxins, such as trichothecenes and zearalenone [14].

### 5.3. Bacteria

Certain lactic acid bacteria (LAB) belonging to* Lactobacillus* and* Leuconostoc* can grow in soft drinks containing fruit juices [93]. LAB have been isolated from fruits, fruit juices, and packaging materials [79]. These bacteria are also resistant to benzoic and sorbic acids. Common LAB responsible for spoilage are* Lactobacillus paracasei, L. brevis, L. buchneri, L. plantarum, L. perolens,* and also* Leuconostoc mesenteroides* and* Weissella confusa* [78]. Depending on the species and growth conditions, sugar catabolism can lead to the formation of lactic acid, ethanol, acetate, succinate, or formate [94]. Formic acid has been proposed as a LAB spoilage indicator in apple juice [95]. Metabolites of LAB are responsible for loss of carbonation and increasing astringency in soft drinks [79]. Some strains produce diacetyl, which gives a buttery taste and smell.* L. mesenteroides* and* W. confusa* are able to produce extracellular polymers composed of fructose or glucose, which are responsible for ropiness in drinks or biofilm formation on technological surfaces [78, 96, 97].

Acetic acid bacteria (AAB) are less common in soft drinks than LAB, since they are strictly aerobic and require at least some oxygen for growth [79]. AAB are acid-tolerant bacteria and grow at pH 3.0 ÷ 3.8, producing acetic, gluconic, lactic, and succinic acids, acetaldehyde, or ketones [79, 98]. The taxonomy of AAB has been modified significantly as new genera have been isolated, and new molecular methods of identification have been introduced [99]. Many AAB tolerate commonly used preservatives (benzoates, sorbates, and dimethyldicarbonate) [98]. Their growth in soft drinks can cause flavor changes, package swelling, ropiness, haze, or sediments. Many species have the ability to form biofilms on production surfaces or packaging materials [78, 100, 101]. Besides the earliest isolated genera* Acetobacter* and* Gluconobacter*,* Gluconacetobacter* and* Asaia* rods are also common contaminants in soft drinks [100, 102–104]. Serious problems with AAB may occur in beverages packaged in oxygen-permeable containers, for example, in certain types of PET bottles.

Coliforms (e.g.,* Klebsiella*,* Citrobacter*, and* Enterobacter*) and other members of* Enterobacteriaceae *are generally acid-intolerant, although some are able to proliferate in juices with pH values below 4.3 [79]. Extracellular polymers maybe also be produced, so Gram-negative bacteria can be detected as specimens of biofilm consortia in plant environments [101, 105–107].

### 5.4. Pathogens

Studies on microbiological quality of carbonated soft drinks have shown that, as a result of poor hygiene, soft drinks can contain high numbers of pathogenic bacteria. Enteric pathogens do not belong to indigenous microbes in fruit. Rather, contamination results from direct or indirect contact with faeces [108]. Bacterial pathogens can remain viable in carbonated soft drinks for different periods [109, 110]. The pathogenic bacteria most commonly encountered in fruit juice-related outbreaks of foodborne disease are enterohemorrhagic or Shiga-toxin-producing* E. coli*, the serotype O157:H7, and various serotypes of* Salmonella* [111].* E. coli* and* Salmonella* have been shown to be capable of surviving up to 48 hours in a cola soft drink, while* Yersinia enterocolitica* have been found to be able to survive in a commercial orange soft drink (pH 3.5) for 3 days at 30°C [112].

Many exotic juices used in modern beverage formulations (e.g., acai, melon, persimmon, and papaya) have low acidity (pH 4.8–6.2). These juices provide conditions suitable not only for the survival but also for the growth of pathogenic bacteria [108]. Such bacteria are able to survive in acidic juices long enough to transmit diseases. Concentrates used for soft drink production may provide a good environment for pathogenic bacteria to survive. For example,* Listeria monocytogenes* and* Y. enterocolitica* have been found to be capable of surviving for lengthy periods in various frozen juice concentrates and in freshly pressed orange juice (pH 6.3) [112, 113]. Incubation at a low temperature (4°C) usually enhances the survival of pathogenic bacteria [114].

Parasites and viruses may also be associated with fruit juice-related disease outbreaks. Protozoa do not replicate outside their hosts, but they can survive for long periods in the environment in a resting stage, that is, in oocysts. The protozoan* Cryptosporidium parvum* has been isolated from apple cider and juice [111]. The survival rates of oocysts in natural mineral waters vary. Waters with high mineral content have been found to have higher inactivation rates at 20°C compared to waters with low mineral content [115].

Viruses do not grow in foods, as they need living cells to replicate. Hepatitis A, norovirus, and rotavirus could potentially transmit diseases via improperly produced beverages. Hepatitis A was transmitted via orange juice during an outbreak in the 1960s [111]. It was shown that the SA11 rotavirus is able to survive for 3 hours at 28°C in fresh papaya (pH 5.1) and honeydew melon (pH 6.3) juices, although not in pineapple juice (pH 3.6). In cold-stored fruit juice (pH 3.01), rotavirus survived for 3 days. Norovirus has been associated with outbreaks traced to raspberries irrigated with contaminated water [14].

### 5.5. Preservation

Acidity is the most important factor in the preservation of soft drinks. Low pH greatly increases the effects of heat treatment while also acting as an additional barrier to the growth of microorganisms. The pH level of most soft drinks is below 4.0. The great majority of heterotrophic bacteria are unable to grow at such low pH values. However, fungal growth is possible at low pH levels. Chilled storage is often used to extend the open shelf life of fruit juices. Most spoilage yeasts and molds are able to grow at 5°C, albeit slowly. Some molds, such as* Penicillium* spp., can grow in chilled juices at 0°C. Lactic acid bacteria gradually lose viability in chilled juices. The use of oxygen-impermeable packaging can double the shelf life of juices from 35 to 65 days. It is often recommended that, after opening, products should be chilled and consumed within 3 days.

Most spoilage incidents caused by yeasts are controlled by preservative systems [13]. However, certain yeast species show resistance, especially to weak-acid preservatives. These strains belong to the fermentative yeast species* Saccharomyces cerevisiae, Schizosaccharomyces pombe, Zygosaccharomyces* spp., and* Dekkera* spp. [14, 75, 78, 108].* Zygosaccharomyces bailii* is able to grow in the presence of both 60% w/v glucose and preservatives far in excess of the legal limits in Europe. Resistance to sorbic and benzoic acids by* Z. bailii* may be caused by reduced uptake combined with metabolism of the preservatives [116, 117]. Other preservative-resistant spoilage yeasts include* Issatchenkia orientalis* (*Candida krusei*),* Pichia membranifaciens*,* Schizosaccharomyces pombe*, and* Saccharomyces cerevisiae* [84].

Certain LAB of* Lactobacillus and Leuconostoc* can grow in soft drinks containing fruit juices [71]. Also new AAB of* Asaia* spp. have been isolated from reclaimed fruit beverages and flavored waters preserved by DMDC, benzoate, or sorbate at concentrations 1.5 mmol/L and higher, limiting the possibilities of preventing spoilage in similar drinks. These bacteria have, additionally, been found in processing equipment in the form of biofilm, which is persistent and difficult to remove by usual cleaning methods [100, 102, 104].

Like common molds, preservative-resistant molds form white, delicate, fluffy, cottony masses suspended in liquids and can grow in limited oxygen. These molds usually grow slowly in bottled beverages and are often detected only after the drinks have reached the marketplace [117].

### 5.6. Pasteurized Drinks

Drinks containing fruit juices, teas, and so forth with no added preservatives are usually pasteurized at temperatures below 90°C. Spoilage is mainly due to the survival of spore-forming bacteria, yeasts forming heat-resistant ascospores, and ascospores of heat-resistant molds belonging to the genera* Byssochlamys spectabilis*/*Paecilomyces variotii*,* Talaromyces* spp.,* Penicillium arenicola,* and* Neosartorya* spp. [14, 79].* Propionibacterium cyclohexanicum* has also been isolated from spoiled pasteurized fruit juices with an off-flavour.* Propionibacterium cyclohexanicum* is a Gram-positive pleomorphic rod that produces propionic, acetic, and lactic acids as products of sugar fermentation. Its growth in orange juice is inhibited by high concentrations of preservatives, potassium sorbate (500 mg/L) and natrium benzoate (1000 mg/L). However,* Propionibacterium* spp. cells are able to survive pasteurization procedures. They are also capable of growing even at refrigerator temperatures [118].

Spore-forming bacteria of the genera* Bacillus* and* Clostridium* are usually inhibited in acid soft drinks. Nonetheless, their spores can remain viable [78]. It is difficult to kill spores completely using conventional heat treatment techniques. For example, heat treatment (sterilization) at 121°C effectively lowers the survival rate of vegetative cells of* Geobacillus stearothermophilus* within a few seconds. However, to have the same effect on spores of* G. stearothermophilus*, the duration of heat treatment must be increased to 10 ÷ 20 minutes [119, 120]. With the development of functional beverages containing fibre and/or vegetable/fruit juices, the importance of spore-forming bacteria as spoilers is increasing [80]. Anaerobic butyrate-forming clostridia such as* Clostridium butyricum* and* Clostridium sporogenes,* which cause a rancid off-flavour in the final product, have been shown to be transmitted by sugar syrups used in the beverage industry. These bacteria can be active even at pH values of 3.6 ÷ 3.8 [14].

Another spore-forming bacteria,* Alicyclobacillus* spp. (ACB), is often associated with the spoilage of soft drinks including carbonated and noncarbonated fruit juices, lemonade, isotonic water, and ice-tea [121]. Among Alicyclobacilli,* A. acidoterrestris* is the primary contaminant. However* A. acidophilus, A. acidocaldarius, A. cycloheptanicus, A. hesperidium, A. herbarius,* and* A. pomorum* have also been detected [122, 123]. Spoilage is usually noticeable by a specific medicinal odor caused by the production of guaiacol and halophenols [121]. Sediment, haze, and discoloration may also appear, although these are comparatively rare [14, 124]. The characteristic musty, mouldy, or earthy off-flavor in pasteurized soft drinks is caused by the metabolites, geosmin, 2-methylisoborneol, and 2-isopropyl-3-methoxypyrazine, produced by* Streptomyces griseus*. These bacteria are able to grow in soft drinks in conditions of limited oxygen and at temperatures as low as 4°C [14, 80].

## 6. Conclusion

Soft drinks consumption is still a controversial issue for public health and public policy. Over the years, numerous studies have been conducted into the possible links between soft drink intake and medical problems, the results of which, however, remain contested. All the ingredients in soft drinks should be approved for use under the appropriate regulations governing the maximal concentrations and acceptable daily doses. Nevertheless, there is a strong body of evidence to support the existence of health risks associated, especially, with overconsumption and with certain artificial colorings and preservatives. Lesser known to the public are risks involving chemical and microbial contamination from containers, production equipment, and (in the case of microbial contamination) certain ingredients themselves. These can affect even the so-called “healthy” low-calorie and functional drinks. Consumers require further education on the use of ingredients, additives, and packaging materials and their potential effects on human health. In turn, producers are under pressure to ensure the health safety of their goods, both from legislation and from consumer demand.

## Figures and Tables

**Table 1:** Types of soft drinks.

Type of soft drink	Description
Bottled water	Potable water, water with flavorings and minerals/vitamins. (i) Still water: noncarbonated, mineral, spring or table water, with or without added flavorings and vitamins/minerals. (ii) Carbonated water: mineral, spring or table water, low carbonated waters, naturally sparkling or sparkling by CO_2_ injection. (iii) Flavored water: unsweetened water, with essences and/or aromatic substances.

Bulk/hot water	Potable water sold in packs of over 10 L for use in dispensers.

Carbonates	Sweetened, beverages with carbon dioxide, syrups for home dilution and out-of-home carbonated soft drinks.

Juice	100% pure fruit or vegetable juice without ingredients, except permitted minerals and vitamins, with sweetening agents (less than 2%).

Nectars	Diluted fruit/vegetable juice and pulp, with sweetening agents, minerals, and vitamins.

Still drinks	Flavored ready-to-drink, noncarbonated beverages, containing fruit or nonfruit flavors or juice content (to 25%).

Squash/syrups	Non-ready-to-drink products, marketed as concentrates for home consumption including fruit and non-fruit-based products and flavors.

Fruit powders	Non-ready-to-drink products in powder form.

Iced/ready-to-drink tea/coffee drinks	Tea-based or coffee-based drinks and non-ready-to-drink powders and liquid concentrates for dilution.

Sports drinks	Products described as “isotonic,” “hypertonic,” or “hypotonic,” still or carbonated, ready-to-drink, or non-ready-to-drink powders and concentrates; also fruit and nonfruit flavored drinks.

Energy drinks	Energy-enhancing drinks, mainly carbonated and containing taurine, guarana, glucose, caffeine, exotic herbs and substances, minerals, and vitamins.

**Table 2:** Examples of quality changes in soft drinks associated with common spoilage microorganisms.

Group	Genera/species	Metabolites	Visual defects	Odours
Yeasts	***Aureobasidium*** * pullulans*; ***Candida*** *davenportii, C. parapsilosis, C. tropicalis, C. solani*; ***Clavispora*** * lusitaniae*; ***Cryptococcus*** * albidus, C. laurentii*; ***Debaryomyces*** *hansenii, D. etchellsii, D. polymorphus*; ***Dekkera*** * anomala, D. bruxellensis*; ***Galactomyces*** *geotrichum*; ***Issatchenkia*** *orientalis*; ***Kluyveromyces*** * lactis, K. marxianus*; ***Metschnikowia*** *pulcherrima*; ***Pichia*** *anomala, P. jadinii, P. membranifaciens, P. subpelliculosa*; ***Rhodotorula*** *glutinis*; ***Saccharomyces*** *cerevisiae, S. bayanus, S. exiguous*; ***Schizosaccharomyces*** *pombe*; ***Zygosaccharomyces*** *bailii, Z. bisporus, Z. lentus, Z. rouxii *	CO_2_, ethanol, diacetyl, acetaldehyde, esters, pentadiene, pectin degradation, EPS	Haze, clouds, surface films, swollen packages	Yeasty, aldehyde, vinegar, pineapple note

LAB	***Lactobacillus*** *acidophilus, L. brevis, L. buchneri, L. paracasei, L. perolens, L. plantarum*; ***Leuconostoc*** *mesenteroides*; ***Weissella*** *confusa *	Lactic acid, CO_2_, ethanol, diacetyl, formic acid, EPS	Loss of carbon dioxide, ropiness, turbidity	Cheesy, sour, green apple

AAB	***Acetobacter*** *suboxydans*; ***Gluconobacter*** *oxydans*; ***Gluconacetobacter*** *sacchari*; ***Asaia*** *lannensis, A. bogorensis *	Acetic acid, gluconic acid, carbon dioxide, ethyl acetate, acetoin, EPS	Haze, ropiness, surface films	Sour, vinegar

ACB	***Alicyclobacillus*** *acidoterrestris, A. acidophilus*, *A*. *acidocaldarius*,* A. cycloheptanicus, A. hesperidium*, *A*. *herbarius*,* A. pomorum *	2,6-Dibromophenol, guaiacol	Without defects	Antiseptic and smoky taints

Molds	***Aspergillus*** *niger, A. penicillioides, A. versicolor*; ***Byssochlamys*** *nivea *(*fulva*); ***Cladosporium*** *sphaerospermum*; ***Fusarium*** *oxysporum*; ***Eupenicillium*** *brefeldianum*; ***Mucor*** *circinelloides*, *M.racemosus*; ***Neosartorya*** *fischeri*; ***Paecilomyces*** *fulva*, *variotii*; ***Penicillium*** *glabrum*; ***Rhizopus*** *stolonifer*; ***Talaromyces*** *flavus* (*macrosporus*)	Formic acid, gluconic acid, increase in pH level (metabolism of acids), gas production, pectin degradation	Mycelial mats, discoloration swollen packages	Musty, stale
